# Comparison of visual field progression in new-diagnosed primary open-angle and exfoliation glaucoma patients in Sweden

**DOI:** 10.1186/s12886-020-01592-w

**Published:** 2020-08-05

**Authors:** Marcelo Ayala

**Affiliations:** Eye Department, Skaraborg Hospital, Skövde, Sahlgrenska Academy, Gothenburg University & Karolinska Institute, 541 85 Skövde, Sweden

**Keywords:** Exfoliation glaucoma, Visual field progression, Open-angle glaucoma, Sweden

## Abstract

**Background:**

The present study aimed to compare visual field progression in new-diagnosed exfoliation versus open-angle glaucoma patients.

**Methods:**

Retrospective study. The study included patients with new-diagnosed primary open-angle and exfoliation glaucoma. All patients were followed for 3 years with reliable visual fields. At least five reliable fields were needed for inclusion. Exfoliation and open-angle glaucoma were defined based on the European Glaucoma Society guidelines. Visual field evaluation was performed using the software threshold 24–2 of the Humphrey Field Analysis. Outcomes: Visual field progression. For visual field progression, three different strategies were used: mean deviation (MD), visual field index (VFI), and the guided progression analysis (GPA).

**Results:**

The study included 128 subjects, of the 54 in the open-angle and 74 in the exfoliation glaucoma group. The MD difference values were higher in the exfoliation (− 3.17 dB) than in the primary open-angle (− 1.25 dB) glaucoma group in the three-year follow-up period. The difference between groups was significant (t-test, *p* = < 0.001). The difference in VFI was calculated for the 3 years follow-up period. The difference was higher in the exfoliation (− 7.65%) than in the primary open (− 1.90%) glaucoma group (t-test, *p* = < 0.001). The GPA showed progression in 58% of cases in exfoliation, and 13% in primary open glaucoma group (Chi-square, *p* = < 0.001).

**Conclusion:**

The present study found a more frequent and faster visual field progression in exfoliation than in primary open-angle glaucoma patients. New-diagnosed exfoliation glaucoma patients must be controlled and treated more strictly than primary open-angle glaucoma patients to avoid visual field deterioration.

## Background

Glaucoma is an optic neuropathy that can lead to blindness. It’s one of the most common causes of blindness in western countries [1]. Loss of ganglion cells with a consequent loss of visual field characterized the disease. Until now, no treatment has been discovered to cure the disease, and its character is progressive. Still, the cause of glaucoma is unknown, but several risk factors have been described. Increased intraocular pressure (IOP) is the most common risk factor [2]. There are different types of glaucoma; among them, the two most common in Scandinavia are primary open-angle (POAG) and exfoliation glaucoma (EXFG) [3].

Primary open-angle glaucoma (POAG) can be defined as a progressive optic nerve disease that can lead to deterioration of visual field [4]. These changes are usually associated with increased IOP. The term “primary” shows no known cause such as trauma, pigmentation, exfoliation, or inflammation for the development of this glaucoma. Risk factors commonly associated with POAG are age, elevated IOP at baseline, lower central corneal thickness, and family history [5–8].

Exfoliation glaucoma is characterized by the presence of a greyish material that deposits in the anterior chamber of the eye. This material is composed of proteins; the origin of the material is unknown. Exfoliation material has been isolated in different parts of the eye. The pupil, the anterior part of the iris, trabecular meshwork, anterior capsule of the lens, and even in other ocular and extraocular tissues [9–12] showed the presence of exfoliation material. The deposits at the trabecular meshwork occlude the pores at the trabecular meshwork diminishing the outflow, thus increasing the IOP.

As was described above, glaucoma is a progressive disease—glaucoma progression estimates by visual field deterioration. According to previous studies [13, 14], progress shows a large variability among individuals. It’s essential to determine the rate of progression in each individual to choose the best treatment modality available; treatment must be individualized. The European Glaucoma Guidelines [4] recommends 5–6 visual fields in 2 years after diagnosing glaucoma to estimate progression. The Swedish Glaucoma Guidelines [15] recommend 5–6 visual fields in 3 years. The approach was judged to be more realistic to the Swedish resources allocation. Still “golden standard” in Sweden for glaucoma progression analysis is visual field testing.

The present study aimed to compare visual field progression in new-diagnosed exfoliation versus open-angle glaucoma patients.

## Methods

The present study was a cohort retrospective chart-review, including all patients with new glaucoma diagnose attending to our Department from 1st January 2012 till 31st December 2015 (4 years). The Ophthalmology Department at the Skaraborg’s Hospital is a tertiary unit taking care to around 250,000 inhabitants. The search was performed in our database based on the international classification of diseases (ICD) codes for glaucoma (ICD-10: H409, H401A, and H401C) combined with a code for the first visit (F).

Exclusion criteria:

1. Wrong diagnosis or insufficient information.
2. Patients that cannot perform reliable visual fields at the beginning and/or at least five reliable visual fields 3 years after diagnosis. Reliable visual fields were defined as: false positives ≤15% and/or false negatives ≤20% and/or fixation losses ≤30%.
3. Patients with advanced glaucoma damage defined as mean deviation (MD) ≥20 dB and/or visual field index (VFI) ≤40%. This criterium was chosen to avoid “floor effects” in which further loss of visual field defects can no longer be detected [16].
4. Patients that required glaucoma surgery during the follow-up period. Uneventful cataract surgery, as well as selective laser treatment (SLT), were not considered as exclusion criteria.
5. Patients were suffering from another eye disease that could alter visual fields under study period like central vein occlusion, retinal detachment, etc.

A detailed medical and ocular history was recorded for each patient. Included patients were referred to the Ophthalmology Department due to high IOP (≥ 21 mmHg) detected by an optician. Visual acuity, intraocular pressure (IOP) measurements, gonioscopy, optic nerve status, visual field examinations, name and amount of medications, and the presence or absence of exfoliation were recorded. A Snellen chart was used to measure visual acuity. The IOP was measured by Goldmann tonometry. To assess trabecular meshwork, gonioscopy was performed in a dark room using a goniolens with undilated pupils. The presence of Sampaolesi’s line was also recorded at the inferior angle. The patient’s pupils were dilated with 2.5% Phenylephrine and Tropicamide 0.5% (Bausch & Lomb UK Ltd., 106 London Road-Kingston-upon-Thames-Surrey-KT2 6TN-England). Eyes were classified as no exfoliation if there was no evidence of exfoliation material on the pupil, lens, or the angle with dilated pupils. The optic nerve status was studied using a 90-D lens, and the average of vertical cupping was recorded as the cup-to-disc ratio. All patients performed repeated Humphrey Field Analysis (Carl Zeiss, Carl-Zeiss-Straße 22, 73,447 Oberkochen, Germany) using the software threshold 24–2. Only reliable visual fields were considered (see above). Eye-drops were measured as the number of medicines (compounds) and not as the number of bottles. If both eyes were suffering glaucoma, one eye was chosen at random.

For visual field progression analysis, three different methods were used. The first method was based on the general parameter called “mean deviation” (MD). This parameter was chosen since several studies still use MD as an indicator of progression [14, 17, 18]. Cataract development can also alter MD. The second method used was the progression analysis based on the “visual field index” (VFI). The device calculated the VFI automatically. The device also performed a regression analysis and expressed if the regression curve (slope) statistically significant deviates from the expected values. The “rate of progression” is calculated as the amount of VFI deterioration (%)/year. This kind of progression analysis is also called as “trend analysis.” The third progression analysis studied was the guided progression analysis (GPA). Also included in the device and performed automatically. The GPA is, as difference with the previous, “an event analysis.” The machine compares every single point to prior examinations. A progression analysis is performed (GPA); the results can be: no, possible or likely progression. For analysis purposes in this study, the results were evaluated as “no progression” or “progression,” which included both “possible” and “likely” progression.

Exfoliation glaucoma was defined as untreated IOP of 21 mmHg or beyond, open anterior chamber angle in gonioscopy, glaucomatous visual field defect (at least two repeatable Humphrey 24–2) and glaucomatous optic nerve damage, concomitant with the presence of exfoliation material, observed at the anterior lens capsule and/or at the pupillary border according to the definition of the European Glaucoma Society [4]. Primary open-angle glaucoma (POAG) was defined in the same manner but without the presence of exfoliation material in a dilated pupil.

The study adhered to the tenets of the declaration of Helsinki Declaration.

### Statistics

For statistical analysis, SPSS (IBM, 1 New Orchard Road Armonk, NY 10504, USA) software was used. The continuous variables (IOP, MD, and VFI) were tested for normality using the Kolmogorov-Smirnov test and for homoscedasticity using the Levene’s test. The continuous variables were tested for significance using the t-test, and the Chi-square test was used for testing non-continuous variables (GPA). The significance level was set at 0.05.

## Results

Totally, 168 patients suffering from newly diagnosed glaucoma were identified in the 4 years recruiting period. Of them, 40 patients were excluded and the rest, 128 patients were included and analyzed.

Excluded cases are shown in Table 1. A greater number of cases were excluded in the exfoliation (*n* = 25) vs. the primary open-angle glaucoma group (*n* = 15). The most common reason for exclusion in both groups was advanced visual field damage at the beginning. This reason accounted for 56% in the exfoliation and 40% in the primary open-angle glaucoma group. Trabeculectomy was the second reason for exclusion. It accounted for 36% in exfoliation and 20% in primary open-angle glaucoma patients.

**Table 1:** Exclusion criteria

Cause	POAG	EXFG
Advanced Visual Field damage (MD ≥ 20 dB and/or VFI ≤ 40%)	6 (40%)	14 (56%)
Trabeculectomy	3 (20%)	9 (36%)
No reliable visual fields	3 (20%)	1 (4%)
Other diseases	3 (20%)	1 (4%)
Total	15 (100%)	25 (100%)

*POAG* primary open-angle glaucoma, *EXFG* exfoliation glaucoma, *MD* mean deviation, *VFI* visual field index

Included patients were 128 subjects, of the 54 in the primary open-angle, and 74 in the exfoliation group. The baseline parameters of the included patients are shown in Table 2. It’s interesting to observe that visual acuity was lower in the exfoliation than the primary open-angle glaucoma group though age was not significantly different. Also, exfoliation glaucoma presented more often unilateral than bilateral (78 vs. 22%).

**Table 2:** Baseline demographic and clinical characteristics on the patients included in the study (*n* = 168)

	POAG	EXFG	Test	P
Age (years) (SD)	69.6 (8.69)	71.4 (6.39)	T-test	0.20
Gender (M/W) (%)	28/26 (52/48)	29/35 (45/55)	Chi-square	0.57
Visual acuity (Snellen) (SD)	0.91 (0.11)	0.84 (0.21)	T-test	0.027*
Phakia/pseudophakia (%)	50/4 (92.5/7.5%)	60/4 (93.75/6.25%)	Chi-square	0.34
Bilateral/unilateral (%)	37/17 (69/31%)	14/50 (22/78%)	Chi-square	< 0.001*
Intraocular pressure (IOP) (mmHg) (SD)	27.62 (5.4)	32.9 (4.81)	T-test	< 0.001*
Mean deviation (MD) (dB) (SD)	−5.57 (4.83)	−5.65 (4.52)	T-test	0.92
Pattern deviation (PSD) (dB) (SD)	5.10 (3.81)	4.41 (3.14)	T-test	0.28
Visual field index (VFI) (%) (SD)	87.49 (12.45)	87.65 (12.65)	T-test	0.94

*POAG* primary open-angle glaucoma, *EXFG* exfoliation glaucoma

(*) = Significant values

Another parameter that differed considerably among the groups was the IOP at the beginning of the study. The average IOP in exfoliation was 32.9 mmHg; meanwhile, it was 27.6 mmHg in the primary open-angle glaucoma patients (t-test, *p* < 0.001).

The mean deviation (MD), pattern standard deviation (PSD), and visual field index (VFI) did not differ among the groups at the beginning of the study. No difference in the studied parameters can be explained by the fact that many patients with advanced visual field defects were excluded in the exfoliation than in the primary open-angle glaucoma group.

The continuous variables: IOP, MD, and VFI were tested for normality and homoscedasticity. All variables were normally distributed according to the Kolmogorov-Smirnov test (IOP: *p* = 0.127; MD: *p* = 0.244; VFI, *p* = 0.182). Regarding homoscedasticity, the Levene’s test also showed equality of variances in all the variables (IOP, *p* = 0.623; MD, *p* = 0.092; VFI, *p* = 0.067).

The results of the study after 3 years follow-up are shown in Table 3. The difference in the mean deviation (MD) between the beginning and end of the study (3 years follow-up) was calculated for both groups. The MD difference values were higher in the exfoliation (3.17 dB) than in the primary open (1.25 dB) glaucoma group. The difference between groups was significant (t-test, *p* = < 0.001).

**Table 3:** Results after three years follow-up

	POAG	EXFG	Test	*P*-value
Average difference MD in 3 years (dB) (SD)	1.25 (1.63)	3.17 (2.26)	T-test	*P* = < 0.001*
Average difference in VFI in 3 years (%) (SD)	1.90 (1.45)	7.66 (5.68)	T-test	*P* = < 0.001*
Average rate of progression VFI (%/year) (SD)	−0.54 (1.07)	−2.17 (2.08)	T-test	*P* = < 0.001*
Progress/No progress VFI (%)	8/46 (15/85)	32/32 (50/50)	Chi-square	*P* = < 0.001*
Progress/No progress GPA (%)	7/47 (13/87)	37/27 (58/42)	Chi-square	*P* = < 0.001*
Average IOP (mmHg) (SD) at the end of the study	15.93 (2.56)	16.34 (2.68)	T-test	*P* = 0.40
Average number of medications (SD)	1.75 (0.84)	2.80 (0.80)	T-test	*P* = < 0.001*
Number of SLT/ No SLT (%)	2/52 (4/96)	10/54 (19/81)	Chi-square	*P* = 0.002*
Number of Cataract operation/ No cataract operation (%)	4/50 (7/93)	11/53 (17/83)	Chi-square	*P* = 0.02*

*POAG* primary open-angle glaucoma, *EXFG* exfoliation glaucoma, *MD* mean deviation, *SD* standard deviation, *VFI* visual field index, *GPA* guided progression analysis, *IOP* intraocular pressure, *SLT* selective laser trabeculoplasty

(*) = Significant values

The difference in VFI was calculated for the 3 years follow-up period. The difference was higher in the exfoliation (7.65%) than in the primary open (1.90%) glaucoma group. The difference was statistically significant (t-test, *p* = < 0.001). The device also automatically gave a calculation of VFI deterioration/year (Rate of progression/year). The decrease of VFI/year was 2.17% for exfoliation and 0.54% for the primary open glaucoma group. The difference was also statistically significant (t-test, *p* = < 0.001).

Regarding the proportion of cases that showed progression or not in the trend-based (VFI) showed significant progress in 50% of exfoliation and 15% of primary open glaucoma. The difference was significant (Chi-square, *p* = < 0.001). Similar results were found when checking for the event analysis strategy, guided progression analysis (GPA). Using the GPA, 58% showed progression in the exfoliation, and 13% in the primary open glaucoma group. The difference was significant (Chi-square, *p* = < 0.001).

The average number of medications used at the end of the study was higher in the exfoliation (2.80) than in the primary open (1.75) glaucoma group. The difference was statistically significant (t-test, *p* = < 0.001). The number of SLT treatments performed was also higher in the exfoliation (19%) than in the primary open (4%) glaucoma group. The difference was significant (Chi-square, *p* = < 0.005).

## Discussion

The present study showed a more frequent and faster visual field deterioration among exfoliation than primary open-angle glaucoma patients. The results are probably not surprising; what is new in this study is that all included patients were new-diagnosed as glaucoma. Previous studies [2, 19] measuring visual field progression included both new and old diagnosed patients. The other interesting point to raise is that very few published studies have included exfoliation patients. The significant majority of studies measuring progression included only open-angle glaucoma patients [14, 20, 21].

The results coming from this study are in accord with results coming from the Early Manifest Glaucoma Trial (EMGT) [19] that showed an increased visual field progression in exfoliation than in primary open glaucoma patients. The authors found a progression of MD: − 3.13 dB/year, a higher value than the one found in this study (− 1.05 dB/year). The higher values found in the EMGT can be attributed to the fact that patients were untreated. In the EMGT, the VFI values were not studied; since VFI was not available at the time. Kim et al. [22] published a study identifying risk factors for visual field progression in a retrospective cohort study, including 1317 eyes. They found exfoliation as a risk factor for progression. Visual field progression was MD: − 0.14 dB/year in the POAG and − 0.24 dB/year in the exfoliation group. Regarding VFI, they described a deterioration of − 0.32%/year in the POAG and − 0.59%/year in the exfoliation group. The present study found a progression in the VFI values of − 0.54%/year in POAG and − 2.17%/year for exfoliation.

Interestingly, Kim et al. study included only 4.4% of patients suffering from exfoliation glaucoma. Relative similar results in visual field progression were reported by Kocaturk et al. [23] The authors reported a VFI deterioration of − 0.3%/year in the POAG (*n* = 146) and − 0.43%/year in the exfoliation (*n* = 123) group.

The study had an extended follow-up of 15 years. All the previously mentioned studies showed a difference in progression between the different types of glaucoma. However, Moraes et al. [24] showed different results, calculating exfoliation as a risk factor for progress in a multivariate analysis. Among the included glaucomas, exfoliation glaucoma patients had higher rates of visual field progression than POAG, normal-tension glaucoma (NTG), etc. The authors found a visual field progression of − 0.65 dB/year in MD values. However, the increased risk associated with exfoliation became nonsignificant after adjusting for baseline and intercurrent variables, suggesting the influence of other ocular parameters, such as higher IOP peaks, more common in exfoliation glaucoma patients. These results must be confirmed in further studies. Comparisons among studies are difficult to perform due to differences in follow-up extension, exclusion/inclusion criteria, ethnicity, age, treatments, etc.

The present study showed a faster visual field progression in MD values compared to previous studies. It’s possible to speculate that the difference can be attributed to the fact that all included patients were new-diagnosed glaucoma subjects. After establishing diagnose, the patients were treated, and the visual field deterioration diminished compared with the high progression values the disease has in its natural history (without treatment) [19]. It’s possible to speculate that the disease’s progression slows down with the right treatment after some years. Another possible explanation to the results found in this study can be genetic; all included patients were of Scandinavian origin. Previous studies [25] showed differences in gene expression among different populations studied.

All included patients were sent to the Ophthalmology Department at the Skaraborg’s Hospital by opticians. Opticians referred the patients when they showed high IOP (≥ 21 mmHg). The way the patients came into the Department created a selection bias, but on the other hand, this is the way we got patients in our Department. Patients suffering from exfoliation but with lower IOP were not included in this study because the optometrists did not send them. Exfoliation glaucoma usually presents as a high IOP glaucoma, so the number of patients with normal IOP suffering from exfoliation glaucoma might be quite low.

Though a good IOP reduction due to medication and laser, exfoliation glaucoma patients continued to progress. The average IOP at the end of the study in exfoliation patients was 16.34 mmHg. It seems that even IOP was quite low, the IOP reduction was not enough to stop the visual field deterioration, and bigger efforts might be necessary to slow down the disease.

All patients got an estimated “target pressure” after their glaucoma diagnose. This target pressure is a clinical-based IOP level based on the patient’s age, bilateral or unilateral presentation of glaucoma, visual field damage, etc. In our clinical practice, a normal target pressure is an IOP around 20 mmHg or below when establishing glaucoma. The IOP “target pressure” always re-evaluates after 3 years when we have the data coming from the visual field examinations. Based on this study’s results, it seems that exfoliation glaucoma patients must be considered to need a lower target pressure than POAG patients.

At the beginning of the study, visual fields were similar in both groups though a higher IOP was measured in the exfoliation group. This point must be interpreted with caution. One can think that exfoliation glaucoma with elevated IOP at diagnose has similar visual field deterioration than POAG patients with lower IOP. Similar visual field deterioration at the beginning of the study explains by the fact that advanced visual field defects were excluded to avoid “ceiling effects.” Exclusion due to very advanced visual field defects was more common in the exfoliation than in the POAG group. Exfoliation glaucoma patients showed not only a faster visual field deterioration but also a larger inter-individual variability. Some patients showed nearly no progress; meanwhile, others progressed very fast. Genetic differences can probably explain this difference in disease progression [25].

This study has some limitations. One is its retrospective design. Though medical records were standardized for glaucoma patients, it’s possible for some missing data. Reporting bias is also a common problem in retrospective studies. One of the most significant limitations of the study is the exclusion of very advanced glaucoma subjects. Patients were excluded to avoid “ceiling effects.” Unfortunately, there is still no suitable method to evaluate glaucoma progression in advanced cases. Another limitation is that the study did not include data about anatomical progress, like optical coherence tomography (OCT) or photographs. At the time the study was performed, it was not a common clinical practice to study our glaucoma patients with OCT. Still, there is no consensus about which method is the best to evaluate glaucoma progression [26].

The present study showed a higher amount of unilateral glaucoma patients in the exfoliation (78%) than in the open-angle (31%) glaucoma group. Higher unilaterality is in accord with previously published studies [3, 27]. All patients were examined with dilated pupils, so the risk to miss the presence of exfoliation was quite low. The presence of exfoliation correlated well with high IOP in the affected eyes. Sometimes the difference between eyes was around 20–30 mmHg. No patient “converted” from unilateral to bilateral during the 3 years follow-up period. According to previous studies [28], approximately 38% of patients converted to bilateral in a 10-year follow-up period. The reason for the unilateral expression of the disease is still unknown. Possibly the condition is bilateral but with an asymmetrical phenotypical expression [29]. Genetic studies have been performed showing that possibly local factors at the eye determine the expression or not of the genotype [30].

## Conclusions

In conclusion, using three different strategies for testing visual field progression it was found a more frequent and faster visual field progression in exfoliation than in primary open-angle glaucoma patients. Exfoliation glaucoma patients must be controlled and treated more strictly than primary open-angle glaucoma patients to avoid visual field deterioration.

## Data Availability

The datasets used and/or analyzed during the current study are available from the corresponding author on reasonable request.

## Abbreviations

POAG: Primary open-angle glaucoma
EXFG: Exfoliation glaucoma
MD: Mean deviation
GPA: Guided progression analysis
VFI: Visual field index
IOP: Intraocular pressure
ICD: International classification of diseases
EMGT: Early manifest glaucoma trial
OCT: Optical coherence tomography
